# LbL Nano-Assemblies: A Versatile Tool for Biomedical and Healthcare Applications

**DOI:** 10.3390/nano12060949

**Published:** 2022-03-14

**Authors:** Ana M. Díez-Pascual, Abbas Rahdar

**Affiliations:** 1 Universidad de Alcalá, Facultad de Ciencias, Departamento de Química Analítica, Química Física e Ingeniería Química, Ctra. Madrid-Barcelona, Km. 33.6, 28805 Alcalá de Henares, Madrid, Spain; 2 Department of Physics, Faculty of Science, University of Zabol, Zabol 538-98615, Iran; a.rahdar@uoz.ac.ir

**Keywords:** layer-by-layer, polyelectrolyte, nano-assembly, electrostatic interactions, polysaccharides, proteins, drug delivery, tissue engineering

## Abstract

Polyelectrolytes (PEs) have been the aim of many research studies over the past years. PE films are prepared by the simple and versatile layer-by-layer (LbL) approach using alternating assemblies of polymer pairs involving a polyanion and a polycation. The adsorption of the alternating PE multiple layers is driven by different forces (i.e., electrostatic interactions, H-bonding, charge transfer interactions, hydrophobic forces, etc.), which enable an accurate control over the physical properties of the film (i.e., thickness at the nanoscale and morphology). These PE nano-assemblies have a wide range of biomedical and healthcare applications, including drug delivery, protein delivery, tissue engineering, wound healing, and so forth. This review provides a concise overview of the most outstanding research on the design and fabrication of PE nanofilms. Their nanostructures, molecular interactions with biomolecules, and applications in the biomedical field are briefly discussed. Finally, the perspectives of further research directions in the development of LbL nano-assemblies for healthcare and medical applications are highlighted.

## 1. Introduction

**Figure 1:**
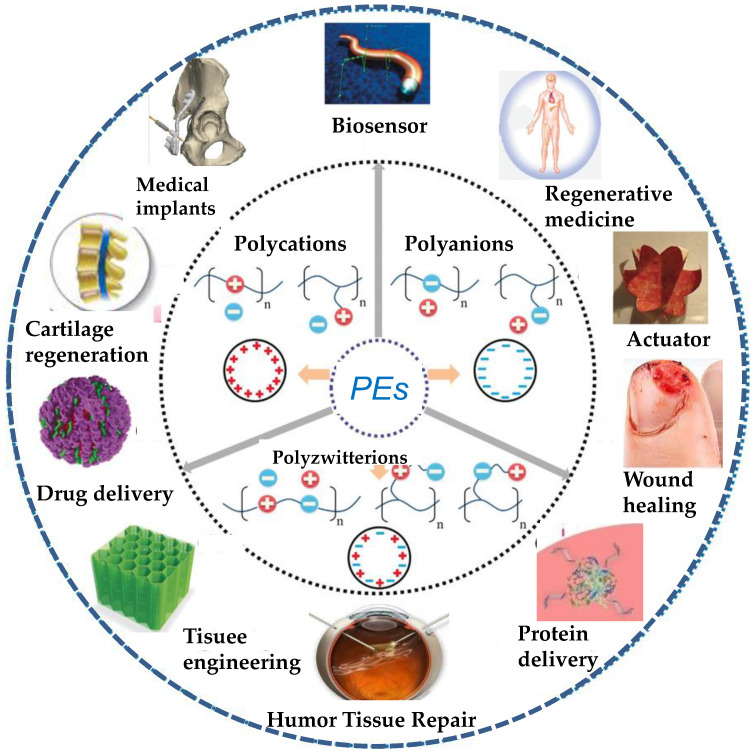
Schematic representation of the biomedical applications of PEs.

Polyelectrolytes (PEs) are a singular kind of polymers with ionizable groups (electrolytes) in each of their repeating units that dissociate in aqueous solutions, making the polymers charged [1]. This behavior can affect surface properties and interactions. Many biological molecules, such as polypeptides, glycoproteins, glycosaminoglycans, and DNA, are PEs. The presence of several types of interactions (electrostatic, hydrophobic, H-bonding, charge transfer, etc.) between polymer chains can have applications in numerous arenas, particularly in the biomedical field, such as drug delivery, protein delivery, tissue engineering, humor tissue repair, biosensors, actuators, regenerative medicine, cartilage regeneration, wound healing, medical implants, and so forth (Figure 1).

PEs can be classified into weak and strong [2]. A “strong” PE is one that dissociates completely in solution for most pH values, while a “weak” one will be partially dissociated for most pH levels, and thus will not be fully charged in solution, and the number of charges can be modified by changing the solution pH, counter-ion concentration, or ionic strength. Their conformation in dilute solution and in the absence of any salt is fully extended, but it can be easily tuned by changing the ionic strength of the medium. Besides, PEs can be polyanionic, polycationic, and polyzwitterionic (Figure 1). Polyzwitterions are defined as formally charged neutral ampholytic polymers containing ionic groups of opposite sign [3], commonly on the same pendant groups. Thus, equal amounts of cationic groups (mostly ammonium) are combined with anionic sulfonates, phosphates, or carboxylates on the same repeat unit to give polyzwitterionic sulfobetaines, phosphobetaines, or carboxybetaines. Poly-ions of opposite electrostatic charges can interact to form polyelectrolyte complexes (PECs), properties of which in aqueous media are conditioned by several factors, e.g., nature, size, charge density, position of ionic groups, size, concentration of macromolecules, ratio of opposite charges, ionic strength, pH, temperature of the medium, etc. [4].

Depending on their shape, PEs are classified into spherical and rigid rod PEs. For instance, globular proteins are spherical, while poly(p-phenylene) is rigid rod-shaped. Besides, based on their architecture, that is, the location of ionic sites, they are named as linear, branched, and crosslinked PEs. Different branched structures are stars (regular and irregular), comb-like, H-shaped, and dendrimers. According to their composition, there are two main types: homopolymers, prepared by crosslinking only one type of monomer, and copolymers by crosslinking more than one type of monomer [5].

**Figure 2:**
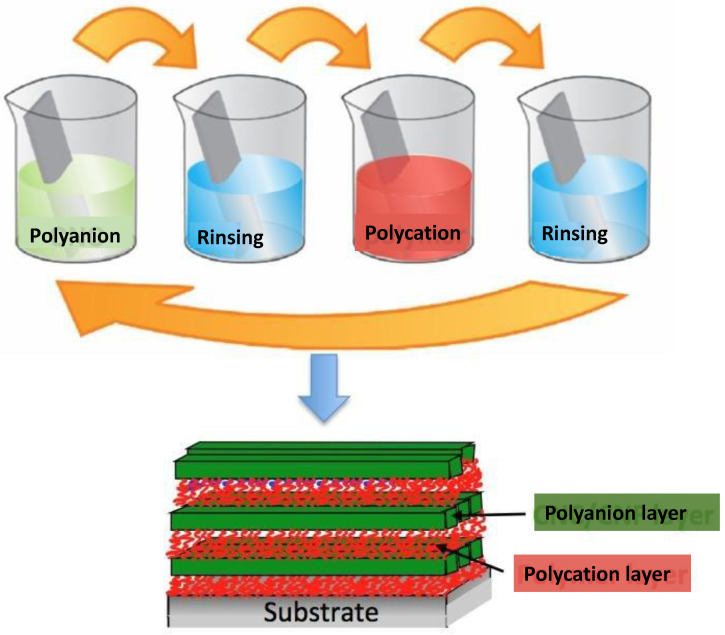
Diagram of the LbL deposition process based on the alternating exposure of a charged substrate to solutions of positively and negatively charged polyelectrolytes.

On the other hand, among current developments in nanotechnology are the preparation of nanofibrous scaffolds, nanocomposite biomaterials, nanohydrogels, laser-fabricated nanostructures, and surface functionalization at the “nanoscale”, which is a successful approach to attain suitable biological responses via specific interactions between the bulk material and a surface coating at the interface [6]. Thus, the need for improving the interface properties in a controllable means encouraged researchers to deposit single layers of organic molecules at the surface. In the literature, numerous techniques to functionalize biomaterial surfaces have been reported, the most important being Langmuir–Blodgett deposition [7] and the self-assembled monolayer method [8]. However, they have some disadvantages, such as the cost, long processing time, limited range of biomolecules, and lack of film stability under physiological conditions, which limit their applications in the biomedical field. In this regard, the layer-by-layer (LbL) assembly method is one of the most common approaches for fabricating thin films in the biomedical field. This method, proposed by Decher and coworkers in 1991 [9], is based on the alternating exposure of a charged substrate to positively and negatively charged solutions of PEs (Figure 2). In order to remove the excess PEs and to prevent the cross-contamination of solutions, after each deposition step, the substrates are rinsed in a washing solution, typically distilled water. Strong PE layers (with high surface charge density) are not significantly altered by rinsing of the LbL assembly since the layer is held by strong interactions. However, weak PEs (with low surface charge density) may be stripped off, restraining successful LbL assembly. In this regard, different techniques, such as zeta potential, quartz crystal microbalance, dynamic light scattering, fluorescence correlation spectroscopy, etc., have been used to monitor the LbL process [10,11]. Consecutive dipping of the adsorbed films into the corresponding PE solutions guarantees a moist environment, improving chain flexibility and ionization during the adsorption stages, and thus thinner and less compact films are usually formed due to a higher grade of adsorption.

The thickness and porosity of the films can be tailored by adjusting the experimental parameters, such as pH, ionic strength, temperature, and polyelectrolyte concentration [10]. The importance of the ionic strength of the solutions can be elucidated taking into account its effect on the solvent quality, and hence on the effective charge density of the PEs due to their role on the charge screening, which causes the transition of the PE conformation from rod-like to a more coiled one as the ionic strength increases due to the reduction of the intra-chain repulsions. Thus, the multilayer thickness increases with the ionic strength.

The influence of the pH can be explained similarly to the ionic strength. In this case, the drop in the strength of the electrostatic interactions occurs due to the effective reduction of the ionization degree of the chains [12]. Generally, the decrease in the ionization degree reduces the solubility of the building blocks, thus promoting their deposition onto the substrate, and hence increasing the multilayer thickness. Besides, the nature of the building blocks plays a fundamental role in the control of the assembly of the films since their choice influences the interactions involved in the process.

The temperature also has a critical role in controlling the solubility of the building blocks, and in some cases dominates over the effect of the ionic strength and the pH level. For instance, it can modify the growth of strong PEs such as poly(diallyldimethylammonium chloride) (PDADMAC) and poly(4-styrene sulfonate of sodium) (PSS) multilayer systems from linear to exponential [12].

This technique permits to design homogeneous coatings with accurate and predictable architecture. Though it was initially developed onto flat, rigid, solid substrates [9], it has been extended to other types of substrates with different natures, shapes, and sizes, such as colloidal particles [5], nanogels [13,14], liposomes, or vesicles [6]. Thus, Caruso et al. [15] and Donath et al. [16] assembled PEs onto spherical particles via this approach, which led to an assembly freely suspended in water. This was carried out by applying LbL coatings onto colloidal particles, which can then be removed to obtain hollow capsules as vehicles for encapsulating various drugs [17]. Besides, these nano-capsules with a solid PE shell are significantly more stable compared to micelles or liposomes [18]. This technique affords a high loading of the therapeutic due to the high surface area of the porous particle and is applicable to a series of materials of different sizes, from proteins to low molecular weight drugs. Sacrificial templates, such as inorganic nanoparticles [19,20], carbon nanotubes [21], quantum dots [22], etc., can be removed using conditions that do not significantly affect the activity of the loaded therapeutic, forming capsules with high loadings. Besides, it is inexpensive, environmentally friendly, and all the deposition stages can be performed under mild conditions.

Multilayered LbL thin films can be designed with controlled structures and properties, such as magnetic, optical, and biological [23]. Taking into account that LbL assembly is usually carried out at room temperature and in aqueous solution, it is appropriate to preserve the activity of proteins, nucleic acids, and other biomacromolecules. Further, it allows to coat structures with nanometer coatings. Besides, in biomedical applications, LbL can be used to attain high levels of biocompatibility both in vitro and in vivo, and an adjusted nanostructure can be used to: (1) adjust the surface topography, (2) load a huge range of biomolecules, as well as organic and inorganic nanomaterials, and (3) incorporate drugs in separate sets of layers for a sequential controlled delivery.

Since the first paper reported by Decher et al. in 1991 [9], millions of articles have been published regarding the LbL approach, and thousands of them are published every year in the biomedical field. In the following sections, some representative examples of LbL assemblies designed for biomedical applications will be described, with the aim to provide the reader with a general overview of the topic.

## 2. Biomedical Applications of LbL PE Nano-Assemblies

LbL nano-assemblies have been used for a wide range of biomedical applications, in particular for drug delivery, protein delivery, tissue engineering, and wound healing, and representative examples are briefly described below. 

### 2.1. Drug Delivery

Drug encapsulation typically results in a lessening of the dose and the contraindications of an administered drug. In this regard, the use of nanomaterials as drug vehicles provides many benefits, including controlled drug release, targeted delivery, and the ability to bypass the cellular surface multi-drug-resistance mechanism [24]. The LbL technique has been used to fabricate drug carriers: by tailoring the location of the therapeutics either in the core or in the different layers, the drug release can be temporally and spatially well-tuned [25]. Nanostructured materials show several advantages, including higher surface area or specific size effects that result in different pharmacokinetics or biodistribution. Thus, designed nanocarriers can exhibit sustained and controlled release at a specific location over an extended period of time [26]. By tailoring the molecular structure on the nanocarrier surface, targeted drug delivery can be attained. These nanostructured materials can be used to treat various kinds of illnesses, such as thromboses, heart disease, diabetes, and even several types of cancer [27].

Suitable drug-delivery nanocarriers should comprise enough functional groups or a reaction site that interacts with the drug or targeting molecule. They can be synthesized by modifying the surface with ligands that are organ- or cell-specific based on the biological targets through ligand-receptor or antibody-antigen interactions [28]. During the last few decades, different shapes of selective recognition carriers for nanomedicine applications targeting cancer cells appeared, with improved therapeutic responses both in vitro and in vivo [29]. Several types of targeting drug-delivery systems, such as immunoglobulin-directed, integrin-directed, transferrin-directed, low-density lipoprotein-directed, and folic acid receptor-targeting, are under intense investigation [30]. Nonetheless, to attain the best therapeutic effects, the nanocarriers should transport the optimal drug amount to the desired target and it should be released at the best rate for a specified time.

**Figure 3:**
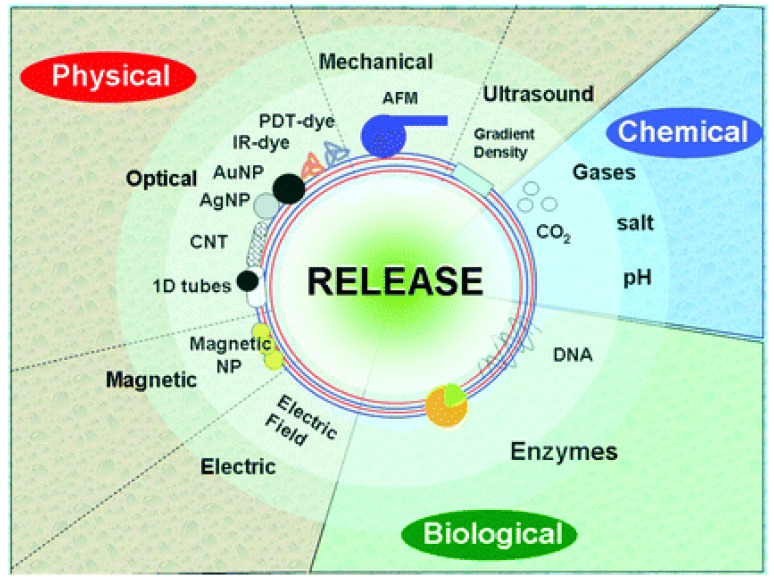
Scheme showing the different types of stimuli for release of encapsulated compounds from LbL materials. Reproduced from [31], with permission from The Royal Society of Chemistry.

The key features of the LbL approach are multifunctionality and responsiveness to a stimuli. These can be divided into three categories [31] (Figure 3): physical (optical, electric, magnetic, mechanical, and ultrasound), chemical (pH, salt, gases), and biological (enzymes and receptors). Using these stimuli, numerous functionalities of nano-shells have been demonstrated: encapsulation, release including that inside living cells or in tissue, sensors, enzymatic reactions, enhancement of mechanical properties, and fusion [32]. The drug release kinetics is influenced by the solubility and the size of the drug, the number and thickness of the LbL layers, and the type of polymers used in the LbL process [33]. By controlling the permeability via parameters such as pH, ionic strength, thickness, nature, and molecular weight of the PEs, the drug release rate can be tailored.

Despite a lot of effort, fulfilling all the above-mentioned requirements remains a big challenge, and very few works have succeeded, as will be discussed below. Two key questions remain, still under thorough investigation [34]: passive targeting strategies via the enhanced permeability and retention effect and active targeting strategies with stimuli-sensitive nanocarriers. Thus, efficient loading of the drug remains a key issue. Loading by diffusion results in a large fraction of the drug lasting in the dispersion medium, often leading to high losses and increased costs. In addition, the characteristics of the internal structure of PE multilayers and the arrangement of PEs within them is not yet clear. Such research is required for understanding the mechanisms of interaction between the encapsulated subject and PEs. Additionally, it is necessary to predict PEs’ interactions in the medium, for instance, the adhesion of microcapsules with the surface, its immunogenetics, and so forth. 

**Figure 4:**
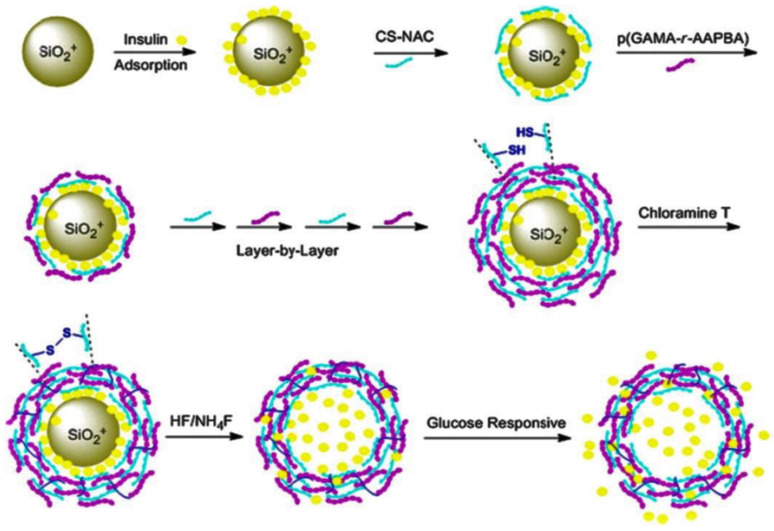
Preparation of glucose-responsive multilayer particles via the LbL process. Reprinted from [35], with permission from Springer Science and Business Media.

Many approaches have been investigated to encapsulate drugs into PE capsules, such as Guo and coworkers [35], who designed glucose-responsive multilayered particles: SiO_2_ cores were joint to insulin and subsequently covered with a coating comprising a chitosan derivative (CS-NAC) and a glycopolymer, p(GAMA-r-AAPBA) (Figure 4). Upon dissolution of the core with NH4F/HF, the nanoparticles were loaded with insulin, which was released based on the glucose concentration and pH value.

**Figure 5:**
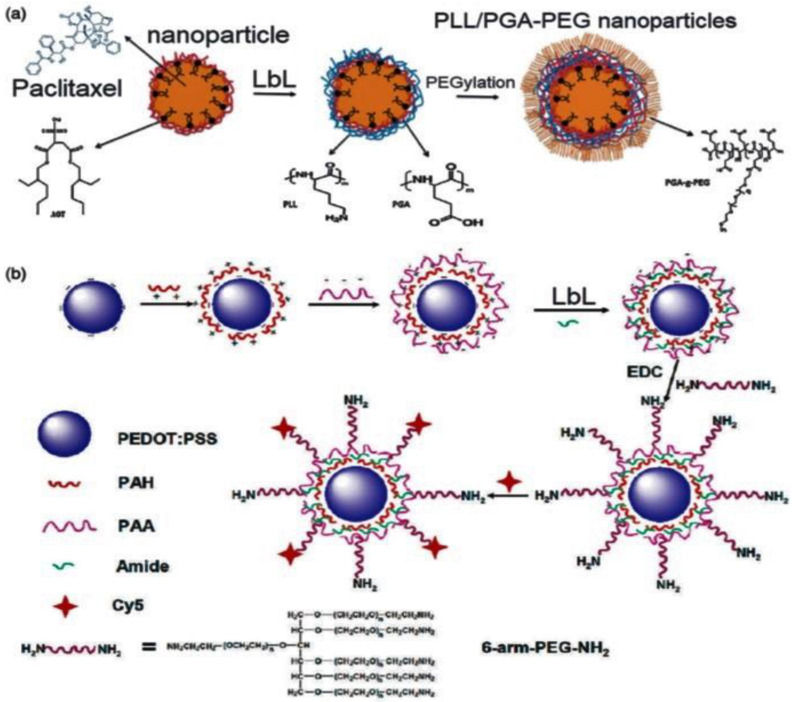
(**a**) Representation of the LbL assembly of PEGylated PLL/PGA nanoparticles for controlled delivery of paclitaxel. Reprinted from [36], with permission from Elsevier. (**b**) LbL assembly of the PEDOT/PSS-PEG nanoparticles for targeted delivery of antitumor drugs. Reprinted from [37], with permission from the American Chemical Society.

LbL-assembled nanoparticles have a strong tendency to accumulate in tumor tissues owing to an improved permeability and retention effect, thus facilitating the targeted delivery of anticancer drugs with enhanced efficiency. Besides, LbL-assembled nanoparticles with the encapsulated therapeutics can flow through the blood for a longer period (i.e., over 4 days [25]). In this regard, PEGylated poly-l-lysine (PLL)/poly-L-glutamic acid (PGA) nanoparticles, with an average size of 100 nm, have been loaded with paclitaxel, a chemotherapy drug used to treat a variety of cancers (Figure 5a) [36]. The nanoparticles were stable in cell culture medium containing serum. The adsorption of the PEGylated PEs extended the durability of the nanoparticles in the blood stream and improved the in vivo tumor-targeting property, thus increasing antitumor efficacy against two cell lines: the mouse colon carcinoma cell line CT26-CEA and the mouse mammary carcinoma cell line 4T1. Given their effectiveness, these nanoparticles may serve as carriers for other chemotherapeutics. Similarly, poly(3,4-ethylenedioxythiophene)/PPS nanoparticles have been LbL-coated with PAH and PAA and further modified with an aminated PEG-derivative via esterification reaction using ethyl-dimethyaminopropyl-carbodiimide (EDC) as a coupling agent (Figure 5b) [37]. The resulting nanoparticles were used for controlled release of antitumor drugs, and showed high stability under physiological conditions. Besides, upon intravenous injection, they displayed a stealth-like behavior together with long durability in blood, resulting in an outstanding tumor uptake. 

Natural polysaccharide/protein biopolymers are interesting targeted drug-delivery systems that can enhance the therapeutic action and reduce the side effects of drugs. In this regard, carboxymethyl cellulose (CMC) and casein (CA)/folic acid (FA) nanogels loaded with curcumin, a natural antioxidant, were prepared by the LbL method for skin cancer treatment [38]. Cellular uptake and cytotoxicity studies revealed that these hybrid nanogels have higher cellular uptake compared to their counterparts without FA, leading to improved cytotoxicity and being apoptotic against MEL-39 melanoma cancer cells. Besides, they displayed higher swelling and drug release at acidic pH, and their blood compatibility was confirmed by in vitro hemolysis. The developed system is suitable for treatment of melanoma or other skin cancers, in which a high selective uptake of curcumin via the transdermal route will increase effectivity.

Supramolecular photosensitizers with LbL assembling capacity have recently emerged as a new strategy to build versatile theranostic nanoplatforms for targeted tumor therapy [39]. In this regard, negatively charged cyclodextrin-grafted hyaluronic acid (HA-CD) and meso-tetra (4-sulfonatophenyl) porphine (TPPS4), a water-soluble tetrapyrrolic dye, were alternatively deposited onto mesoporous silica nanoparticles loaded with tirapazamine (TPZ), a bio-reductive prodrug that is just cytotoxic to hypoxic cells [40]. Subsequently, a paramagnetic resonance imaging agent (Gd^3+^) was hosted via chelating with TPPS4, forming a LbL-coated nano-theranostic platform (TPZ@MCMSN-Gd^3+^) that could actively target tumors with overexpressed CD44 receptor due to synergistic effects.

A drug-delivery system for loading photosensitizer drugs was recently developed via LbL of PSS and PAH onto hollow CaCO_3_ nanoparticles [41]. Phthalocyanine derivatives loaded in the microcapsules preserved their photophysical behavior after encapsulation. The hollow multilayered microcapsules were studied by steady-state and time-resolved techniques, and their biological activity was evaluated in vitro with cancer cells, showing 80% cell death compared to the control.

Poly(lactic-co-glycolic) acid (PLGA)-based nanoparticles are also very good candidates for drug delivery due to their biocompatibility and biodegradability. Many works have investigated the LbL functionalization of PLGA nanoparticles to attain improved drug release efficiency. For instance, Zhou and coworkers [42] LbL-coated chitosan and alginate on PLGA nanoparticles to investigate the antifouling properties against a model protein, albumin. Furthermore, folic acid was covalently bonded to the LbL assembly via carbodiimide chemistry. The multilayer assembly showed little interaction with albumin, corroborating the antifouling characteristics of the coating.

**Figure 6:**
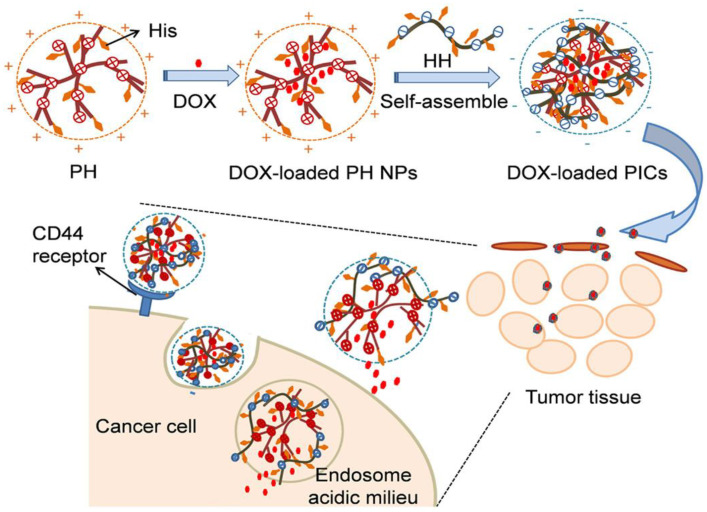
Representation of the synthesis of HH/PH LbL assembly and the pH-controlled targeted drug delivery. Reprinted from [43], with permission from Elsevier.

LbL assemblies of the hyaluronic acid–L-histidine conjugate (HH) and the polyethylenimine–L-histidine conjugate (PH) at different weight ratios have also been developed for the controlled release of doxorubicin (DOX), a small molecular drug [43] (Figure 6). The His moieties reduced the charge density of PEI, hence rendering it biocompatible. The optimum formulation was found at a weight ratio of 4:1, with a drug encapsulation efficiency of 58%. The nanoparticles had a spherical shape, with an average diameter of 170 nm and a zeta potential of −27.2 mV. Drug release was found to be higher at an acidic pH, and showed very good efficiency against B16F10 tumor cells, ascribed to the combination of the tumor-targeting capability of HA and the pH reversibility of histidine moieties. The in vitro cytotoxicity study against B16F10 and NIH3T3 cells revealed that these nano-assemblies had no significant cytotoxicity in both tumor and normal cells. Besides, the assemblies showed good long-term stability since they preserved the size and the drug release profile over 5 days.

**Figure 7:**
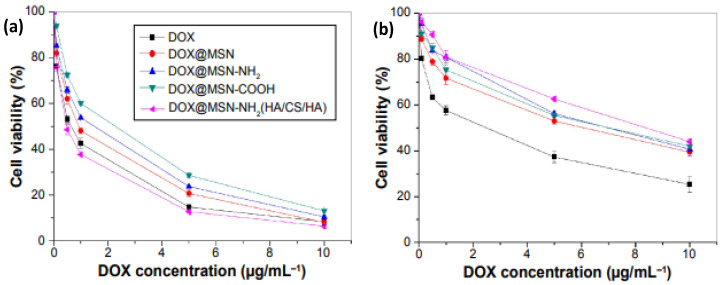
In vitro cytotoxicity assay curves of DOX, DOX@MSN, DOX@MSN-NH2, DOX@MSN-COOH, and DOX@MSN-NH2 (HA/CS/HA) on HepG2 (**a**) and L02 cells (**b**). Nomenclature: DOX: doxorubicin; CS: chitosan; HA: hyaluronic acid; MSN: mesoporous silica nanoparticle. Adapted from [46], with permission from Dove Medical Press Ltd.

Mesoporous silica nanoparticles coated with different types of PEs have also been developed for targeted drug delivery [44]. Their large surface-to-volume ratio, biocompatibility, and versatility allow the encapsulation of a wide variety of therapeutic compounds inside their pores by a simple diffusion mechanism. Upon particle loading, these are clogged using different coating systems or gatekeepers to temporarily limit drug release until the carrier reaches its target tissue, producing a drug burst release effect [45]. In this regard, folic acid-modified CS, which recognizes the folate receptor expressed on the tumor cell surface, and a CD44 receptor-targeted polysaccharide HA were assembled onto silica nanoparticles [46]. The ability of the LbL nanoparticles for pH-triggered load discharge was investigated in vitro using DOX as a model drug. Two pH levels were chosen (7.4 and 5.0) to simulate the blood and tumor environment, respectively. The modified nanoparticles were targeted to cancerogenic regions by specific recognition of the FA receptor or CD44 receptors on the tumor cell surface. Once the nanoparticles were taken up by the cancer cells, DOX was intracellularly liberated from the acid-sensitive membrane in the tumor cell environment. The biocompatibility of bare and DOX-loaded nanoparticles was evaluated in L02 and HepG2 cells with the MTT assay (Figure 7). For all the concentrations, L02 cells displayed higher viability in the presence of DOX@MSN-NH_2_(HA/CS/HA), demonstrating that the former cells are more sensitive to membrane-controlled nanoparticles. The membrane specifically recognized the cancer cells but not normal ones, aided to reduce the drug leakage, and improved the stability of the nanoparticles in the transmission process. 

Versatility is a very important advantage of the LbL technique. It enables to prepare hybrid composite materials combining PEs and organic or inorganic nanoparticles. For instance, the adaptable luminescence of quantum dots (QDs) can be applied in QD-containing PE-based capsules as theragnostic agents [47]. For application of the LbL technique, QDs are usually chemically modified with thioglycolic acid or mercaptoacetic acid (MAA), in order to have a negative zeta potential, and then LbL-coated by PEs such as polyallylamine and polyvinyl sulfonic acid [22]. LbL can also be functionalized by carbon-based fillers. The exclusive combination of carbon materials’ properties with the versatility of the LbL assembly allows fabrication of multifunctional nanocomposite materials with improved mechanical, optical, thermal, electromagnetic, and electrochemical properties [48]. For instance, PE/graphene oxide (GO) multilayers have been fabricated via LbL on sacrificial templates, and their mechanical and optical properties were investigated by nanoindentation and NIR absorption spectroscopy [49]. Incorporation of three GO layers between the PE films increased the Young’s modulus and tensile stress of the assemblies by a factor of 5.6 and 2.6, respectively. The developed structures show great potential as delivery systems in biological tissues with remote triggering. The microcapsules modified by zinc phthalocyanine, a photodynamic dye, offered selective therapy after internalization into cancer cells by excitation with a NIR laser. In a similar study, GO-functionalized microcapsules exhibited a photothermal response and enhanced permeability of the shells by NIR laser treatment [50]. Additionally, microcapsules functionalized by single-wall carbon nanotubes were shown to release drugs in response to the NIR laser [51].

On the other hand, microcapsules modified with inorganic nanoparticles such as TiO_2_ [52], Fe_3_O_4_ [53], gold [54], and Pt [55] have also been recently developed and proposed as biomedical theragnostic systems due to photothermal and sensor functionality. PE microcapsules functionalized with magnetite and silica nanoparticles were prepared via silane-type precursor hydrolysis and LbL, and the effect of ultrasonication on the microcapsules was investigated [56]. The sonication parameters that guarantee efficient disruption of their shells in an aqueous medium were determined. Furthermore, PAH/PSS microcapsules containing nanoparticles in the shell were investigated in terms of sensitivity to ultrasonication in dependence on the location of magnetite within the shell, and it was found that capsules with iron oxide nanoparticles were readily ruptured [57].

LbL assemblies of PE multilayer shells on biocompatible halloysite nanotubes have been synthesized for the entrapment, storage, and subsequent release of three drugs: nifedipine (anti-anginal), furosemide (anti-hypertension and diuretic), and dexamethasone (synthetic corticosteroid) [58]. An increase in the release rate of dexamethasone from 7 h release for bare tubes to 30 h for PE-coated tubes was observed. Encapsulation of dexamethasone in the lumen of the PE-coated halloysite offers a new nano-formulation for controlled release of macromolecules, including drugs, biocides, and anticorrosion agents.

### 2.2. Protein Delivery

Proteins are weak PEs with both positive and negative residues on the solvent-accessible surface. With only five residues (Asp, Glu, His, Lys, Arg) comprising a charge near physiological pH, proteins typically have relatively low charge density. PE–protein electrostatic complexation can be used for protein delivery and the development of nano-assemblies in which proteins are real building blocks [59]. For instance, encapsulation of enhanced green fluorescent protein in poly(2-vinyl-pyridinium)-b-poly(ethylene-oxide) (P2VP-B-PEO) was attained through the formation of electrostatic coacervate-core PEO-shell micelles that released the protein at high salt content [60].

The LbL approach has been used to encapsulate proteins into nanoparticles made of natural polysaccharides, chitosan and dextran sulphate, to obtain protein and peptide delivery systems. The amino groups of the SiO_2_ core were used to link a model protein, bovine serum albumin (BSA), and the PE were assembled via LbL. The BSA-controlled release from hollow nano-capsules was successfully performed at pH levels of 1.4 and 7.4 that simulate stomach and blood conditions, respectively [61]. Other authors prepared CaCO_3_ nanoparticles covered with a PE multilayer film made of poly-L-lysine and chondroitin sulphate, another polysaccharide, crosslinked with gluterladehyde, and evaluated the pH influence on BSA loading: the quantity of BSA loaded into the capsules increased by dropping pH from 5.0 to 3.8 [62]. In a similar work, PSS-doped CaCO_3_ nanoparticles LbL-coated with PAH and poly(metacrylic acid) were used for BSA delivery [63]. The BSA loading can be tailored by changing the pH of the medium, due to changes in the PSS–BSA electrostatic interactions: at pH levels below its isoelectric point (4.8), BSA is positively charged, and it was loaded in the nano-capsules due to the strong interaction with negatively charged PSS. At pH levels higher than 4.8, BSA is negatively charged, hence it can be gradually released by progressively increasing the pH level. Moreover, the systems were biocompatible, as corroborated by in vivo studies using mouse fibroblasts.

**Figure 8:**
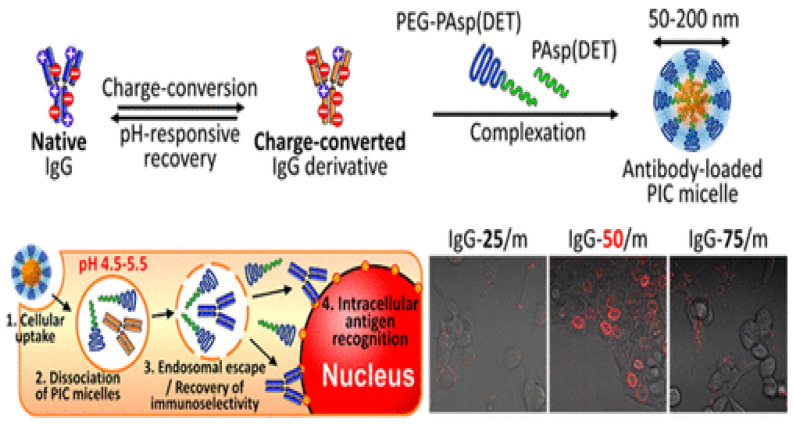
Representation of the formation of IgG-containing PEC micelles (**right**). Observation of in vitro IgG PEC micelle delivery to C26 murine colon carcinoma cells after 24 h incubation. (**left**) Adapted with permission from [65], with permission from the American Chemical Society.

PE micelles as nanocarriers for intracellular delivery of protein therapeutics have also been developed. These micelles have good colloidal stability in vivo and can be tailored to respond to changes in pH, temperature, or in the presence of reducing agents. Such changes permit controlled delivery of different proteins with fine control of the point of release. In the case of temperature responsiveness, a polymer with a physiologically relevant LCST is used to provide sensitivity. For instance, poly[2-(dimethylamino)ethyl methacrylate]-block-poly(glutamic acid) (PDMAEMA-b-p(Glu)) disassembles at basic pH when cooled below the PDMAEMA LCST (40 °C), which has been explored for protein release [64]. On the other hand, pH-responsiveness can be used for effectively delivering antibodies to subcellular spaces by taking advantage of the benefits of polymer shielding, which can protect from loss of affinity and decomposition. Thus, delivery of immunoglobulin G (IgG) antibodies which recognize a family of nucleoporins and enable the direct visualization and quantification of the antigen targeting by following the signal of fluorescent-labeled antibodies on nuclear membranes was performed (Figure 8) using poly(ethylene glycol)-block-poly[N-{N′-(2-aminoethyl)-2-aminoethyl}aspartamide] (PEG−PAsp(DET)) copolymers to form pH-responsive PEC micelles [65]. The IgG was functionalized with citraconic anhydride and converted into a polyanion. The charge-converted IgG micelles were tested for their efficiency in delivering IgG to the nuclear envelope of C26 cells. Micelles were taken into the cell through endocytosis and, at endosomal pH, the citraconic anhydride moieties were cleaved, exposing the original cationic side chains of the protein, leading to localized delivery of IgG to the nuclear envelope, as revealed by fluorescence microscopy (Figure 8). The degree of IgG modification had a significant effect on the rate of the charge reversal and the degree of endocytosis.

pH-sensitive methyl methacrylate (MMA)/itaconic acid (IA) nanogels have been used as carriers to improve the absorption of insulin administered orally. For such purpose, insulin was incorporated into the nanogels via LbL PE complexation [66]. Several parameters, including insulin:nanogel ratio, pH, incubation time, and stirring rate, were optimized. The nano-assemblies were characterized in terms of particle size, polydispersity index, zeta potential, and percent entrapment efficiency. The in vitro release of insulin from the nanogels in the simulated gastric fluid and simulated intestinal fluid were 28.71% and 96.53%, respectively. The nanogels were stable at the 5 °C storage condition during 3 months. The SDS-polyacrylamide gel electrophoresis test indicated that the primary structure of insulin in the nanogels was intact. The oral administration of nanogels to diabetic rats significantly reduced the blood glucose. Therefore, these LbL nanogels can be used as an alternative route for the delivery of insulin.

On the other hand, glycan-functionalized polyethylene glycol-based nanogels have been used for targeted delivery of lectins, proteins capable of specific recognition and of reversible binding to carbohydrate moieties. The nanogels exhibited a wide mesh size and a highly accessible volume, and were found to bind specifically and with high affinity (K_d_ = 0.5–1 µM) to the corresponding carbohydrates [67].

Other groups have considered the use of polysaccharide-based PE nanoparticles for the delivery of insulin. For instance, subcutaneous injection of polyethyleneimine-dextran sulfate nanoparticles led to a more continued response to insulin than injections of free insulin in a diabetic rat model [68]. Additionally, both alginate-based and chitosan-dextran sulfate nanoparticles were developed, which were stable at acidic pH and showed pH-dependent insulin release that could be used to protect the protein in oral delivery applications [69]. Furthermore, the released insulin preserved biological activity up to 24 h, at intestinal pH, and nanoparticles remained stable up to 4 weeks when stored in cold aqueous solutions. The nanoparticles also caused enhanced bioavailability of insulin when delivered orally.

New nanocarriers composed of the glycosaminoglycan hyaluronan (HA) and the polysaccharide chitosan (CS) have also been used for the transmucosal delivery of BSA and the hydrophobic polypeptide Cyclosporine A [70]. Regarding BSA, the highest loading capacity of the nanoparticles (64%) was found for a 0.1:10 HA/CS ratio, which resulted in the smallest particle size, about 320 nm. In the case of the polypeptide, the maximum loading (36%) was also found for the same HA/CS ratio, though the encapsulation was lower due to the hydrophobic nature of the peptide.

**Figure 9:**
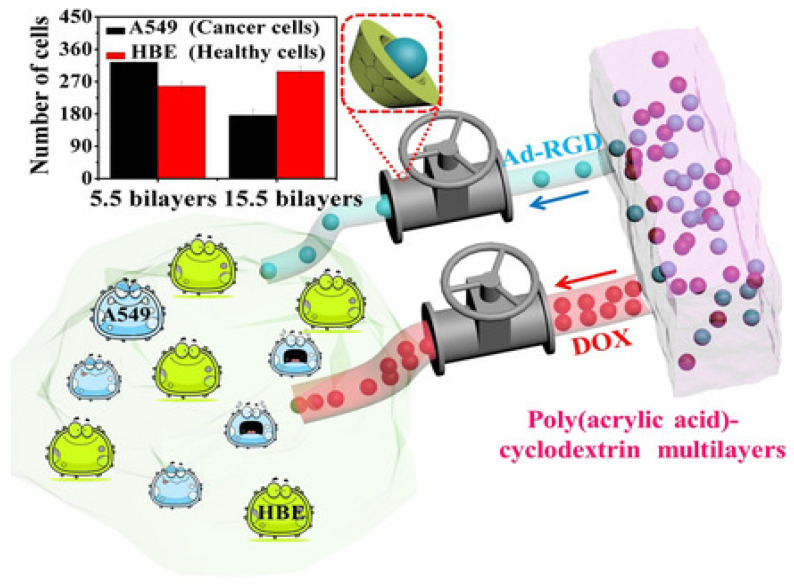
Representation of the selective flux regulation in LbL films by supramolecular interactions between cyclodextrin and its guests. Inset: difference in cell viabilities between human healthy cells (HBE) and cancer cells (A549). Taken from [71], with permission from John Wiley Sons, Inc.

Recently, cyclodextrin (CD) grafted to an anionic PE, poly(acrylic acid) (PAA) LbL-assembled with PAH, has been used for the targeted delivery of adamantane-modified arginine-glycine-aspartic acid (Ad-RGD) tripeptides, and the process was followed by UV/Vis spectroscopy. A control peptide without the Ad head group was used for comparison. For multilayers of (PAH/PAA-CD)n (n = 5.5, 7.5, 10.5, 15.5), the total released amount of the peptide was about 220% of the value of the control one. CDs showed strong, fast, and reversible supramolecular interactions with the tripeptide, that promote molecular loading and slow down diffusion-dependent release. Upon a preliminary study, a maximum difference in cell viabilities between human healthy bronchial epithelial cells (HBE) and cancer cells (A549) was found (Figure 9). Besides, this assembly discriminatively regulates the delivery of bioactive small molecules, such as Ad-RGD and DOX. The proposed methodology could be combined with other treatment strategies, such as targeted delivery or stimuli-responsive delivery, to be used in disease treatment and tissue engineering [71].

Moreover, a novel route that induces nano-porosity within LbL-assembled films has been described for protein delivery. Multilayer thin films were prepared by consecutive adsorption of poly(ethylene imine) (BPEI) polycation, HA polyanion, and cationic gold nanoparticles via electrostatic interactions. The nanoparticles embedded in the assembly structure were simply dissolved using an aqueous cyanide solution to generate the nano-porous film, which resulted in increased loading and release of proteins such as ovalbumin [72].

### 2.3. Tissue Engineering 

**Figure 10:**
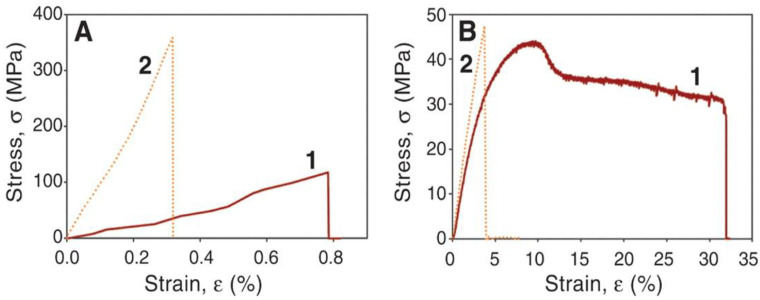
Mechanical properties of PVA/montmorillonite nano-assemblies (**A**) and neat PVA (**B**) without (1) and with (2) glutaraldehyde. Reproduced from [73], with permission from *Science* International.

The LbL technique is a good approach to improve the bioactive properties of synthetic biodegradable polymers used for bone tissue regeneration. In this regard, super-strong and stiff multilayered polymer nanocomposites were prepared via LbL assembly of negatively charged nano-clays (i.e., hectorite or montmorillonite), with poly(vinyl alcohol) (PVA) as a polyanion and poly(diallyldimethylammonium chloride) (PDDA) as a polycation [73]. They obtained nano-assemblies with strong hydrogen bonding interactions between the clay and the polymers that led to outstanding mechanical properties to be used for bone regeneration (yield stress up to 150 MPa and Young’s modulus up to 13 GPa). Besides, some assemblies were crosslinked with glutaraldehyde (GA), leading to uniform stiff and transparent films (Figure 10). 

GA crosslinking augmented the strength, stiffness, and brittleness of both neat PVA and the PVA/clay assemblies. A 3-fold increase in strength compared with the un-crosslinked assembly and a 10-fold rise in comparison with neat PVA were found. The modulus of the crosslinked assembly is comparable to that of Kevlar, in the range of 80 to 220 GPa, and exceeds the stiffness of carbon nanotube-based fibers, ascribed to the covalent bonds that led to a very effective interfacial adhesion between the components. In fact, the observed reinforcement effect was reported to be the combination of a few mechanisms taking place at the nanoscale. The structural order attained via the LbL process maximizes the polymer–nano-clay interactions and restrains chain mobility, resulting in a very efficient load transfer between the polymer phase and the stiff montmorillonite. Moreover, the assemblies displayed excellent stability under humid conditions.

PLGA/hydroxyapatite were assembled with poly(γ-benzyl-L-glutamate) (PBLG) and PLL to develop porous scaffolds that promote cell growth and osteogenic differentiation. The PLL@PLGA/PBLG-g-HA scaffolds were advantageous for cell proliferation and differentiation due to their increased initial cell attachment numbers and enhanced osteogenesis capacity. In vivo digital radiograph evaluation confirmed that the fastest healing was in the bone defects treated with these porous scaffolds when compared with the other groups eight weeks after implantation, and also their mechanical strength was much higher; hence, they are promising candidates for bone defect repair [74].

LbL assembly has also been integrated with various techniques to form functional and antibacterial surface coatings. For instance, poly(dimethyldiallyl ammonium chloride)/poly(styrene sulfonate) and poly(dimethyldiallyl ammonium chloride)/bovine serum albumin microstrips have been prepared with the laminar flow of a microfluidic reactor [75]. With the aim to investigate the integration of LbL assemblies with tissue engineering, glass substrates were coated with nanoparticle/PE layers, and two cell types were used to test the applicability of these coatings for the surface modification of medical implants. Titanium dioxide, silicon dioxide, halloysite, and montmorillonite nanoparticles were assembled with oppositely charged PEs. In vitro cytotoxicity tests of the nanoparticle substrates on human dermal fibroblasts disclosed that the nanoparticle surfaces do not have toxic effects on the cells, which also showed active proliferation on the nanoparticle substrates. Cells deposited on TiO_2_ substrates showed a faster rate of spreading compared with the other types of nanoparticle coatings. Mesenchymal stem cells (MSCs) were also tested. Increasing surface roughness and faster spreading of the MSCs was observed with increasing numbers of layers of TiO_2_. Similarly, tanfloc/heparin PE multilayers on TiO_2_ nanotubes have been developed, and their bacterial adhesion, morphology, as well as biofilm formation were analyzed [76]. Their anti-thrombogenic properties were confirmed via noteworthy reductions in fibrinogen adsorption, Factor XII activation, and platelet adhesion and activation. The modification of the nanotubes with tanfloc/heparin also reduced the adhesion and proliferation of *P. aeruginosa* and *S. aureus* bacteria without biofilm formation. 

Poly(l-lysine)-*g*-poly(ethylene glycol) (biotin)/streptavidin multilayer films led to nano-thin conformal coatings that can be tuned to coat inlets without loss of their viability and function, which is a good approach to adjust the surfaces of living cells and tissues for numerous tissue engineering applications [77]. PE films with alginate, chitosan-graft-phosphorylcholine, and poly-l-lysine-graft-polyethylene glycol were prepared using LbL assembly, and tested for production of universal red blood cells. This is an effective strategy for the design and development of functional multilayers for cell and tissue encapsulation [78].

Recently, free-standing films based on catechol functionalized chitosan, hyaluronic acid, and bio-glass nanoparticles were developed by spin-coating LbL [79]. The incorporation of nanoparticles led to thicker and more hydrophilic films, with rougher surfaces, lower swelling, higher weight loss, and better stiffness. The films showed no negative effects on cellular viability, adhesion, and proliferation, and promoted higher cellular metabolic activity. These LbL nanofilms combine enhanced adhesion with improved mechanical properties and could find applications for hard tissue regeneration membranes. In another study, LbL film coatings were fabricated onto medical implants for osteomyelitis [80]. The antibiotic gentamicin and the osteo-inductive growth factor BMP-2 are weakly positively charged and were used as polycations for preparing multilayered films, along with the negatively charged PAA via electrostatic interactions. The top layer consisted of [Poly1/PAA/GS(gentamycin sulfate)/PAA] tetralayers, which would hinder bacterial biofilms, and the inner layer consisted of [Poly2/PAA/BMP-2/PAA] tetralayers, which would improve bone formation. The developed strategy improved bone-implant interfacial strength 15-fold when compared with prostheses without coating. This study corroborates the potential of this layered release strategy to bring about long-lasting next-generation implants.

Nanostructured 3D assemblies based on chitosan and chondroitin sulphate multilayers by combining LbL technology and template leaching have been developed for cartilage tissue engineering [81]. The attained 3D scaffolds displayed a high porosity and a water uptake capacity of 300%, and could preserve the chondrogenic phenotype and chondrogenic differentiation of bone marrow-derived stromal cells, revealing their potential for use in the field of tissue engineering. Studies on 3D scaffolds based on PE nano-assemblies are still scarce due to the big challenges. More research in this field needs to be carried out in the future.

### 2.4. Wound Healing

**Figure 11:**
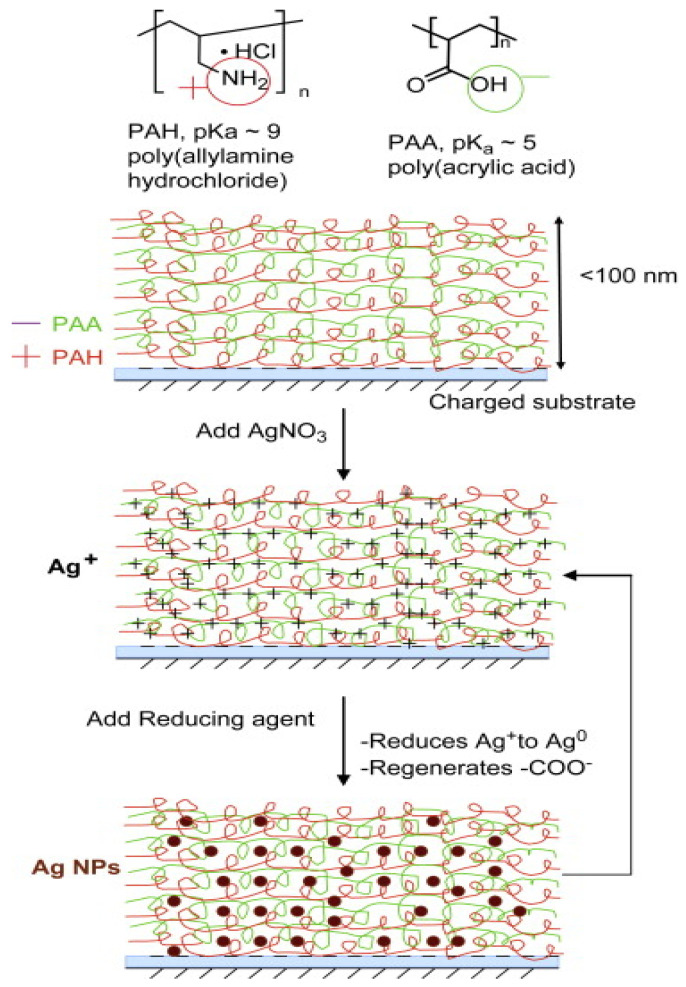
Representation of LbL deposition of multilayers of PAH/PAA on a charged substrate. Post-assembly, the multilayer is incubated with a solution of AgNO_3_. Subsequently, silver ions are reduced to silver nanoparticles. Reproduced from [83], with permission from Elsevier.

Wound dressing materials have the aim of protecting the injured tissue from mechanical trauma and accelerating the healing process. A simple method for producing transparent antimicrobial coatings based on PDDA/PSS LbL multilayers was prepared via dipping in AgNO_3_ in order to incorporate Ag ions, which were reduced into Ag nanoparticles with NaBH_4_ solution [82]. The antibacterial efficiency versus *E. coli* and durability was improved with the nanoparticles. A similar approach was also applied by other groups [83], who loaded Ag nanoparticles in films coated with poly(allylamine hydrochloride) (PAH) and poly(acrylic acid) (PAA) (Figure 11). The Ag^+^ ions were incorporated into the LbL by ion exchange with the acidic protons of PAA, and subsequently reduced to Ag^0^. Since the number of free carboxylic acid groups of PAA that were available for ion exchange with Ag+ was controlled by varying the pH of the PAA solution, the loading of silver within LbL assembly can be finely tuned. Besides, no interactions of mammalian cells with the silver nanoparticle LbL assembly nor cytotoxicity were observed. 

In another work [84], antibacterial coatings were prepared via deposition of negatively charged biocompatible gold nanoparticles (50 nm size) and positively charged lysozyme on porous electrospun cellulose nanofibrous mats. The antibacterial bioactivity of the uncoated and LBL-coated cellulose nanofibers was tested versus *E. coli* and *S. aureus*. Uncoated cellulose did not show an antibacterial effect, while LbL-coated ones displayed strong antibacterial activity, which was enhanced by increasing the number of bilayers.

Analogously, multilayers by covalent LbL assembly of PEI were prepared using terepthalaldehyde as a crosslinker, followed by in situ reduction of Ag ions [85]. The antibacterial efficiency of the LbL assemblies in the presence and absence of Ag nanoparticles was tested, and it was found that the incorporation of the nanoparticles increased antimicrobial activity towards *S. aureus* and *E. coli* up to 99% inhibition due to the release of ions in the aqueous solution. Another PE suitable for wound dressing applications is polyhexamethylene biguanide (PHMB), a polycation antimicrobial with effects against Gram-positive and Gram-negative bacteria, fungi, and human viruses. A LbL nanocoating on a polycaprolactone (PCL) fiber surface with an LbL self-assembled PHMB and PAA coating has been developed, that displayed outstanding antibacterial ability and no toxicity on mammalian cells, suitable to improve wound healing. 

Antibacterial polyhexamethylene guanidine hydrochloride (PHGH) and anti-adhesive heparin (HP) have been successfully coated onto antibacterial polyacrylonitrile nanofibrous membranes via LbL assembly. They exhibited strong antibacterial activities against *S. aureus* and *E. coli*, easy-cleaning properties, and good stability under physiological conditions, thus making them suitable as wound dressing coatings [86]. Another work developed free-standing nanometer size films of chitosan and alginate through a combination of LBL assembly and spin-coating [87]. It was found that the mechanical properties of the nanosheets influenced the PE thickness. An optimal sheet of 75 nm thickness, a critical load of 9.1 × 10^4^ N m^−1^, and an elastic modulus of 9.6 GPa were used for the minimally invasive repair of a visceral pleural defect caused by wound repair. Thus, these nanostructured LbL assemblies are new therapeutic tools for overlapping tissue wounds without the need for conventional surgical interventions. 

## 3. Conclusions and Outlook

LbL can be used to deposit uniform nanometer-scale-thick multilayers from PE solutions. During the LbL assembly process, different types of interactions (electrostatic, hydrophobic, H-bonding, charge transfer, etc.) between PEs lead to the film growth. Since this method can be applied to various charged substances, there is a huge option of available biomaterials, including proteins, nucleic acids, polysaccharides, and so forth. The layering structure, component selection, and surface nature (including biocompatibility, degradability, and size/dimension) can be finely adjusted. The assembly can be performed in aqueous solution under mild ambient conditions, using simple tools. The versatility, simplicity, and inexpensiveness of this promising technique has enabled PEs to cover a comprehensive range of medical applications, including controlled drug and protein delivery, wound healing, tissue engineering, and regenerative medicine. In particular, the ability of LbL films to incorporate a wide variety of drugs, including small molecules and biological drugs, and simultaneously preserving drug activity and the possibility of actively triggered microscale release to localized regions, is particularly interesting, opening the door to new therapeutic approaches in modified implants, wound healing and remediation, cardiovascular devices, and so forth. Besides, the LbL method can be combined with top-down microfabrication and nanofabrication procedures such as photolithography, electron beam lithography, micro-contact printing, ink-jet lithography, and other nanopatterning techniques for a wide range of purposes. 

Introducing nanoparticles of different natures into PE matrices enables to synthesize multifunctional nanocarriers with controllable and tailorable parameters in response to external stimuli (i.e., pH, ionic strength, temperature, ultrasonic treatment, and electromagnetic fields). Numerous works have confirmed the potential of making nanocarriers for theranostics based on a combination of sensing properties with responsiveness towards external stimuli of different natures. Besides, alternated dip-coating is easy to execute and can be straightforwardly automatized. However, some disadvantages of adding nanostructures can also be recognized. Firstly, adding nanostructures increases the expenses and may extend the time necessary for the LbL assembly (in which molecules have to be deposited, to diffuse at the interface, to adsorb, and to find their equilibrium conformation). Thus, process parameters should be optimized to attain quicker and more stable multilayers to enable long-term storage. In this regard, different tests have been carried out to avoid the intermediate rinsing steps and increase the deposition speed. For instance, alternation of spray deposition and spin-coating is a good alternative to deposit layers in a few seconds. In addition, potential biocompatibility issues need to be addressed for some specific types of nanoparticles. For example, researchers have found that silver nanoparticles can have a toxic effect on cells, suppressing cellular growth and multiplication and causing cell death depending on concentrations and duration of exposure. Nanoparticles larger than 200 nm caused an increase in DNA damage in the human cells at concentrations higher than 100 μg mL^−1^ [88].

Taking into account the above-mentioned potential for applying LbL nano-assemblies to in vivo delivery and biomedicine, the main challenges to be addressed in the future are: (1) Fully biocompatible and biodegradable nanomaterials should be synthesized, and their cytotoxicity should be tested. Novel inorganic and organic nanomaterials with different dimensions, shapes, and sizes should be developed and their potential assayed. (2) Additional fundamental studies need to be carried out to understand the interactions between LbL nano-assemblies and biological systems in order to elucidate how these condition the biological responses. (3) More attention should be placed on local delivery applications and the interactions with the local cellular and protein environment. (4) Automatization of the preparation of LbL assemblies should be accomplished, since this is crucial to attain reproducibility and scalability up to an industrial level. Thus, the huge potential is still limited to small-scale research and requires technological and methodological innovation. (5) Compatibility with crossover technologies from other fields, including dipping, de-wetting, roll-to-roll, centrifugation, creaming, spinning, spraying, electrodeposition, magnetic assembly, electrocoupling, microfluidics, etc., is necessary to expand this approach to a wider range of applications in the future, and perhaps even revolutionary. Such combinations with other assembly technologies should also aid to accelerate and automate the assembly process. (6) The use of functional substrates will enable to combine the benefits of different multilayers in a synergistic mode. Overall, the future of LbL nano-assemblies is brilliant, and shows great potential for innovation and application in the healthcare and medical arena.

## Data Availability

Data sharing is not applicable as no new data were created or analyzed in this study.
